# Decreased expression of hypoxia-inducible factor 1α (HIF-1α) in cord blood monocytes under anoxia

**DOI:** 10.1038/s41390-022-02193-7

**Published:** 2022-07-29

**Authors:** Christiane Schlegel, Kai Liu, Bärbel Spring, Stefanie Dietz, Christian F. Poets, Hannes Hudalla, Trim Lajqi, Natascha Köstlin-Gille, Christian Gille

**Affiliations:** 1 Department of Neonatology, Tübingen University Children’s Hospital, Tübingen, Germany; 2 Department of Neonatology, Heidelberg University Children’s Hospital, Heidelberg, Germany

## Abstract

**Background:**

Infections are a major cause for morbidity and mortality in neonates; however, the underling mechanisms for increased infection susceptibility are incompletely understood. Hypoxia, which is present in inflamed tissues, has been identified as an important activation signal for innate immune cells in adults and is mainly mediated by hypoxia-inducible factor 1α (HIF-1α). Fetal tissue pO_2_ physiologically is low but rises immediately after birth.

**Methods:**

In this study, the effect of low oxygen partial pressure (pO_2_) on HIF-1α expression and its targets phagocytosis, reactive oxygen species (ROS) production and vascular endothelial growth factor (VEGF) secretion was compared in vitro between immune cells from adult peripheral blood and cord blood using anoxia, HIF-1α stabilizer desferroxamin (DFO) and *E. coli* as stimuli.

**Results:**

We show that anoxia-induced HIF-1α protein accumulation, phagocytosis, ROS-production and VEGF-expression were greatly diminished in cord blood compared to adult cells. *E. coli* led to HIF-1α gene expression in adult and cord blood immune cells; however, cord blood cells failed to accumulate HIF-1α protein and VEGF upon *E. coli* stimulation.

**Conclusions:**

Taken together, our results show a diminished activation of cord blood immune cells by low pO_2_, which might contribute to impaired reactivity in the context of infection.

**Impact:**

- Neonatal immune cells do not accumulate HIF-1α under low oxygen partial pressure leading to decreased phagocytosis and decreased ROS production.
- We demonstrate a previously unknown mechanism of reduced activation of neonatal immune cells in the context of an inflammatory response.
- This could contribute to the increased susceptibility of newborns and preterm infants to infection.

## Introduction

Infections are one of the most important causes of perinatal morbidity and mortality.^1^ Newborns and especially preterm infants are particularly susceptible to infections, which is primarily attributed to the “neonatal” state of the immune system.^2^ Before birth, the main challenge of the fetal (and maternal) immune system is to prevent mutual rejection. Immediately after birth, neonates are exposed to a myriad of commensals and pathogens, and the immune system must learn to tolerate or fight them off. The mechanisms regulating postnatal adaptation processes of the immune system are only scarcely understood.

Reduced partial pressure of oxygen (pO_2_), i.e., hypoxia, is an important activation signal for adult immune cells during inflammation.^3^ Various immune functions like phagocytosis, cytokine secretion, adhesion, migration and survival are stimulated by hypoxia in adult immune cells.^4,5^ One of the most important transcription factors mediating the effects of hypoxia at the cellular level is the hypoxia-inducible factor 1 (HIF-1).^6^ HIF-1 is a heterodimer complex composed of an alpha subunit (HIF-1α) and a beta subunit (HIF-1β).^3,7^ HIF-1α protein is regulated by pO_2_, whereas HIF-1β is constitutively expressed. Under normoxic conditions, HIF-1α is hydroxylated by prolylhydroxylases (PHDs) and continuously degraded in an oxygen dependent manner.^8^ Under hypoxia, oxygen is missing as cofactor and PHD activity is reduced leading to HIF-1α accumulation. HIF-1α dimerizes with HIF-1β, translocates to the nucleus and binds to hypoxia responsive elements (HRE) in target genes leading to increased transcription of several genes involved in metabolism, angiogenesis, invasion and cell survival.^9,10^ In addition to hypoxia, HIF-1α can also be activated by inflammatory pathways, such as NK-κB.^11,12^

The fetus develops in an environment with low pO_2_, comparable to that found in inflamed tissue.^13,14^ Immediately after birth the pO_2_ rises to adult values. While the effects of hypoxia on adult immune cells have been well studied,^5,15^ little is known about how a reduced pO_2_ affects the functions of cord blood immune cells and research has mainly focused on polymorphonuclear leukocytes.^16^ In the present study, we tested the hypothesis that the chronic hypoxic environment in which the fetus develops influences the response of neonatal immune cells to hypoxia. For this purpose, we stimulated cord blood mononuclear cells (CBMC) and adult mononuclear cells (PBMC) with anoxia and the HIF-1α stabilizer deferoxamine and analyzed HIF-1α expression and innate immune functions like phagocytosis and ROS-production as well as the expression of the HIF-1 target protein vascular endothelial growth factor (VEGF). We show that decreased upregulation of phagocytosis and ROS production in CBMCs compared to PBMCs is caused by an impaired ability to accumulate HIF-1α and thereby describe a previously unknown mechanism for the altered response of neonatal immune cells to inflammatory stimuli, which may underlie the increased susceptibility of neonates to infection.

## Methods

### Patients

Cord blood was collected from healthy term neonates (≥37 + 0 gestational weeks) immediately after primary Cesarean section. Children with intra-amniotic infection (defined by the German Society for Gynaecology and Obstetrics (DGGG) as maternal fever (≥38.0 °C), increased maternal inflammatory markers without any other cause (CRP > 10 mg/l or elevation of white blood cell count >15000/μL), fetal or maternal tachycardia, painful uterus and foul-smelling amniotic fluid) were excluded. Parents gave written informed consent and the study was approved by the local ethics committee (458/2019BO1). The cord blood samples were collected anonymously, so that no statements can be made about sex, exact gestational age, etc. Peripheral blood from healthy volunteer donors was collected as control.

### Cell isolation and culture

Mononuclear cells (MNC) from heparinized cord blood (CBMC) and peripheral blood (PBMC) were isolated by density gradient centrifugation according to a previously described protocol.^17^ Heparinized whole blood was diluted in phosphate-buffered saline (PBS) to a total volume of 35 ml and added carefully onto 15 ml lymphocyte separation solution (Biochrom GmbH, Berlin, Germany). Cells were centrifuged and the MNC layer was collected. Cell count was determined in a SYSMEX-KX21 cytometer (Sysmex GmbH, Norderstedt, Germany) and cells were diluted in RPMI 1640 (Pan Biotech, Aidenbach, Germany) supplemented with 10% fetal calf serum (FCS), 1% penicillin/streptomycin and 1% glutamine. For western blot analyses cells were set to a final concentration of 4 × 10^6^ cells/ml and seeded to 6-well-plates (5 ml/well). For functional analyses, cells were set to a final concentration of 2 × 10^6^ cells/ml and seeded to 12-well- or 24-well-plates (2 respectively 1 ml/well).

For anoxic cell culture, plates were placed into an airtight plastic cylinder with an anaerobic gas producing bag (Thermo Scientific™ Oxoid™ AnaeroGen™ 2.5l-bag, Thermo Fisher Scientific Inc., Waltham, USA) for 4 h.

For desferroxamine (DFO) stimulation, cells were treated with 10 µM DFO in RPMI 1640 supplemented with 10% FCS, 1% penicillin/streptomycin and 1% glutamine (Sigma Aldrich, Taufkirchen, Germany) for 4 h.

### In vitro infection model

A clinical isolate of *E. coli* K1, carrying the green fluorescent protein (*gfp*)-mut2 encoding plasmid pCD353, expressing a prokaryotic variant of GFP controlled by a lactac promoter (*E. coli*^GFP^),^18^ or the corresponding wild-type strain were grown on agar plates, supplemented with either kanamycin (50 g/ml; Sigma-Aldrich, Steinheim, Germany) and Isopropyl-β-D-thiogalactopyranosid (IPTG, 1 mmol/l, Sigma) for GFP induction or without supplements. After 16 h, a single colony was picked and grown in Lennox L broth-medium (Invitrogen, Karlsruhe, Germany) with and without supplements until early logarithmic growth.

To analyze the phagocytic capacity, CBMC and PBMC were stimulated with *E.coli*^GFP^ at a multiplicity of infection (MOI) of 1:50 for 1 h. Bacteria were removed by centrifugation through a FCS-cushion. Phagocytosis of *E.coli*^GFP^ by monocytes was determined by flow cytometry.

### Flow cytometry

Antibodies used for extracellular staining of monocytes and their surface molecules were purchased from BD Pharmingen, Heidelberg, Germany (CD14-PE (clone M5E2) and Annexin V) and from Miltenyi Biotec, Bergisch Gladbach, Germany (CD11b-PE (clone REA731), CD14-APC (clone TÜK), CD18 (clone TS1/18)). All antibodies were tested for their specificity by isotype control staining, when introduced to our laboratory. Data acquisition was performed with a FACSCanto II flow cytometer (BD Bioscience) and data were analyzed via FlowJo V10 (FlowJo, LLC, Ashland, OR). To assess the expression level of GFP, Rhodamine, CD11b and CD18 on monocytes, we used the geometric mean fluorescence intensity (gMFI).

### Reactive oxygen species (ROS) detection

For detection of ROS, 1 × 10^6^ PBMCs and CBMCs were incubated with dihydrorhodamine 123 (DHR 123, Sigma Aldrich) in PBS for 5 min in a water bath at 37 °C. Next, cells were stimulated for 10 min with 60 ng/ml of phorbol myristate acetate (PMA, Sigma Aldrich). Then, cells were washed, stained with anti-CD14-APC and ROS production was analyzed by flow cytometry.

### Protein isolation and western blot

For protein isolation cells were stimulated for four hours with anoxia or *E. coli*, harvested on ice and washed in ice-cold PBS. 100 µl/4 × 10^7^ cells of lysis buffer (50 mM HEPES pH 7.4, 1% Triton X-100, 150 mM NaCl, 1 mM EDTA, 1 mM PMSF, 1% Protease inhibitor cocktail (Roche Diagnostics, Mannheim, Germany)) was added and cells were snap-frozen in liquid nitrogen. Protein lysates were centrifuged to remove cell debris and samples were frozen at −80 °C until further analysis. Protein concentration was determined by bicinchoninic acid assay (BCA-assay, Thermo Fisher Scientific, Waltham, MA).

40 µg of total protein were separated by 10% SDS-PAGE and transferred to polyvinylidenfluoride (PVDF) membranes (Merck Millipore, Burlington) with a wet blotting apparatus (40 V, 200 mA and 150 W for about 16 h, BioRad, Feldkirchen, Gemany. Membranes were blocked in 5% nonfat milk/PBS/0.1% Tween for 1 h and immune-blotted for 24 h with primary antibodies (mouse anti-human HIF-1α 1:500 (cat. No. 610959), BD Biosciences; rabbit anti-human PHD2 1:500 (NB 100–137), Novus Biologicals, Centennial, USA; horseradish peroxidase (HRP)-conjugated secondary antibody (goat anti-mouse (sc-2005) or goat anti-rabbit (sc-2004) conjugated to Horseradish Peroxidase (HRP), 1:1000, Santa Cruz Biotechnology) was diluted in 5% nonfat milk/PBS/0.1% Tween 20 and the blots were developed by a chemiluminescence reaction using Odyssey® Fc Imaging System (LI-COR Biosciences, Lincoln or iBright CL1000 Imaging System, Thermo Fisher Scientific). Densitometric analysis of protein bands was performed with the ImageJ software (National Institutes of Health, Bethesda, MD).

### RNA isolation and qRT-PCR

For HIF-1α qRT-PCR, PBMC and CBMC were stimulated for 48 h with anoxia or *E. coli*, harvested on ice and washed with ice-cold PBS. Cell pellets were frozen at −80 °C until further analysis. Total RNA was extracted using the NucleoSpin RNA Mini kit (Macherey Nagel, Düren, Germany) following the manufacturers’ instructions. RNA concentration and quality were measured in a NanoDrop™ 2000 spectrophotometer (Thermo Fisher Scientific).

For cDNA synthesis, 300 µg RNA were applied. After addition of random hexamer primers (Thermo Fisher Scientific), samples were heated to 65 °C for 5 min followed by cooling down to 4 °C. Then, a master mix (consisting of fivefold reaction buffer, ribolock RNase inhibitor, dNTPs and reverse transcriptase, all from Thermo Fisher Scientific) was added and the samples were heated again (25 °C for 5 min, 42 °C for 60 min, 70 °C for 5 min). The final cDNA-concentration was calculated to 50 ng/µl. At the end, samples were diluted with HPLC water in a 1:1 ratio. Real-time qRT-PCR reaction was performed using a LightCycler® 480 Instrument II (Hoffmann-La Roche AG, Basel, Switzerland). Prefabricated primers for HIF-1α (unique assay ID: qHsaCEP0050075), EGLN1 (unique assay ID: qHsaCEP0058182), and RPL37A (unique assay ID: qHsaCEP0052538) were purchased from Bio-Rad Laboratories.

RPL37A was used as house-keeping gene and data were evaluated via ΔΔCt-method.

### VEGF-ELISA

For VEGF ELISA, PBMC and CBMC were stimulated for 16–22 h with anoxia or *E. coli*. Afterwards, supernatants were collected and stored at −80 °C until further analysis. ELISA was performed using the Human VEGF DuoSet ELISA and DuoSet Ancillary Reagent Kit 2 (both from R&D Systems, Minneapolis, United States of America) following the manufacturer’s instructions. For each sample duplicates were measured and the mean of the optical density was converted into the amount of VEGF in pg/ml based on the values derived from the standard curve.

### Statistics

**Fig. 1:**
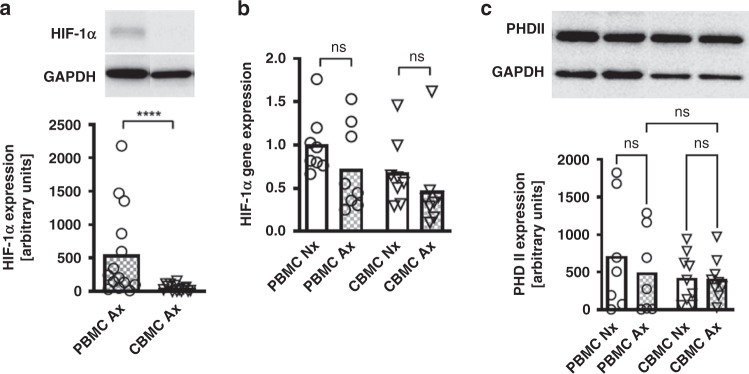
HIF-1α expression in cord blood and adult mononuclear cells under anoxia. Peripheral blood mononuclear cells (PBMC) and cord blood mononuclear cells (CBMC) were isolated and cultured under normoxia or under anoxia for 4 (**a**) or 48 (**b**) hours. Expression of HIF-1α was analyzed on protein level via western blot and on RNA level via PCR. **a** Representative western blot shows expression of HIF-1α and housekeeping gene GAPDH under anoxia in PBMC (left line) and CBMC (right line). Bar plot shows HIF-1α protein expression under anoxia in western blot assessed by densitometry in PBMC and CBMC. Bars represent pooled data from 14 independent experiments and standard deviation is depicted. ****p < 0.0001; Wilcoxon matched-pairs signed rank test. **b** Dot plot with bars shows HIF-1α mRNA expression under normoxia and anoxia in PBMC and CBMC relative to the housekeeping gene RPL37A assessed by qPCR. Bars represent pooled data from 6 independent experiments and mean is depicted. ns not significant; Wilcoxon matched-pairs signed rank test. **c** Representative western blot shows expression of PHD II and housekeeping gene GAPDH under normoxia and anoxia in PBMC (left two lines) and CBMC (right two lines). Dot plot with bars shows PHD II protein expression under normoxia and anoxia in western blot assessed by densitometry in PBMC and CBMC. Bars represent pooled data from 7 to 9 independent experiments and mean is depicted. ns not significant; Kruskal–Wallis test.

Statistical analysis was performed by GraphPad Prism version 9.1.2. Values were tested for Gaussian distribution using D’Agostino and Pearson omnibus normality test. As values were not normally distributed, differences were determined using the Wilcoxon matched-pairs signed rank test. We used a paired statistical test because we know that the reactions of immune cells to hypoxia are extremely dependent on the environmental conditions and the duration of the stimulation, as well as the processing after hypoxia exposure and thus stimulated always an adult donor in parallel with an umbilical cord blood donor. When more than two groups were compared, the Kruskal–Wallis test was applied (Fig. 1c). A *p* value <0.05 was considered statistically significant.

## Results

### Decreased HIF-1α accumulation in cord blood mononuclear cells under anoxia

First, we analyzed the protein expression of HIF-1α in CBMC in comparison to PBMC after four hours of culture under anoxia. Mononuclear cells cultured under normoxia accumulated no HIF-1α protein. Anoxic culture led to accumulation of HIF-1α in PBMC, but only to a marginal expression in CBMC (relative expression 552.5 ± 670.6 versus 52.8 ± 49.3, *n* = 14, *p* < 0.001) (Fig. 1a). A similar effect was observed when PBMC and CBMC were stimulated with the alternative HIF-1α stimulus *E. coli* (Supplementary Fig. 1A). On transcriptional level, anoxic culture did not induce HIF-1α gene expression neither in PBMC nor in CBMC (Fig. 1b), while stimulation with *E. coli* strongly induced HIF-1α gene expression in PBMC and to a lesser extend also in CBMC (Supplementary Fig. 1B). Protein expression of PHD2 did not differ between normoxic and anoxic culture or between PBMC and CBMC (Fig. 1c). There were also no changes in PHD2 protein expression after stimulation with E.coli (Supplementary Fig. 1C).

### Increased phagocytosis activity in adult monocytes but not in cord blood monocytes under anoxia

**Fig. 2:**
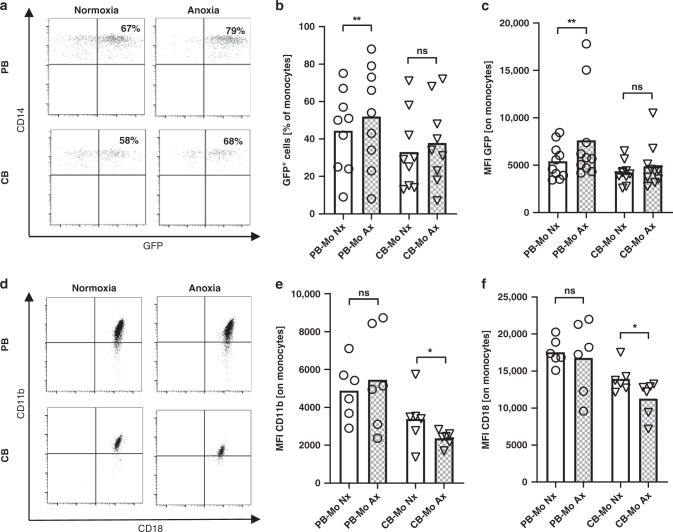
Phagocytosis rates and expression of phagocytosis receptors of cord blood and adult monocytes under anoxia. Peripheral blood mononuclear cells (PBMC) and cord blood mononuclear cells (CBMC) were isolated and cultured under normoxia or under anoxia for four hours. **a**–**c** Afterwards, GFP-expressing *E. coli* (*E. coli*-GFP) were added to the culture for 1 h. **a** Representative density plots show the expression of GFP in CD14^+^ adult monocytes (upper plots) and cord blood monocytes (lower plots) in the upper right quadrant after culture in normoxia (left plots) or anoxia (right plots). **b**, **c** Dot plots with bars show the percentages of GFP expressing monocytes (**b**) and the mean fluorescent intensity (MFI) for GFP expression in monocytes (**c**) after culture in normoxia (blank bars) or in anoxia (checked bars). **d** Representative density plots show the expression of CD18 (*x*-axis) and CD11b (*y*-axis) on CD14^+^ adult (upper plots) and cord blood (lower plots) monocytes. **e**, **f** Dot plots with bars show expression of CD11b (**e**) and CD18 (**f**) on monocytes cultured under normoxia (blank bars) and under anoxia (checked bars). Bars represent pooled data from 6 to 10 independent experiments and mean is depicted. **p < 0.01, *p < 0.05, ns not significant; Wilcoxon matched-pairs signed rank test.

Monocyte phagocytosis activity is a pivotal innate immune effector function and has been shown to become increased in adult monocytes under hypoxia in a HIF-1α dependent manner.^19^ Thus, we analyzed phagocytosis of PB and CB monocytes under normoxic and anoxic conditions. Representative dot plots are presented in Fig. 2a. In PB monocytes, percentage of monocytes taking part in phagocytosis was increased under anoxia compared to normoxia (44.4 ± 21.6% vs. 52.0 ± 27.0%, *n* = 9, *p* < 0.05), while in CB monocytes there was only a marginal and not significant increase in phagocytosis rate (33.0 ± 20.4% vs. 37.9 ± 21.8%, *n* = 9, n.s.) (Fig. 2b). Likewise, the amount of ingested *E. coli*-GFP increased in PB monocytes under anoxic conditions (MFI GFP 5415 ± 1826 vs. 7613 ± 4785, *n* = 9, *p* < 0.05) while in CB anoxia had no significant effect (MFI GFP 4208 ± 1177 vs. 4892 ± 2266, *n* = 9, n.s) (Fig. 2c). To check whether the differences in phagocytosis under anoxia between PB and CB monocytes may be mediated by altered expression of phagocytosis receptors, we next analyzed expression of CD11b and CD18 on PB and CB monocytes under normoxia and anoxia. Interestingly, we found no differences in CD11b or CD18 expression on PB monocytes after culture under normoxic and anoxic conditions (MFI 4876 ± 1515 versus 5456 ± 2645 for CD11b and 17,550 ± 1813 versus 16,778 ± 4941 for CD18, *n* = 6, n.s.) but a decreased expression of both CD11b and CD18 on CB monocytes under anoxia (3384 ± 1413 versus 2365 ± 340 for CD11b and 13,948 ± 1908 versus 11,285 ± 2451 for CD18, *n* = 6, *p* < 0.05) (Fig. 2d–f). Apoptosis rates were not different between culture under normoxia or anoxia and between PB and CB monocytes (Supplementary Fig. 2).

### Increased production of reactive oxygen species in adult monocytes but not cord blood monocytes under anoxia

**Fig. 3:**
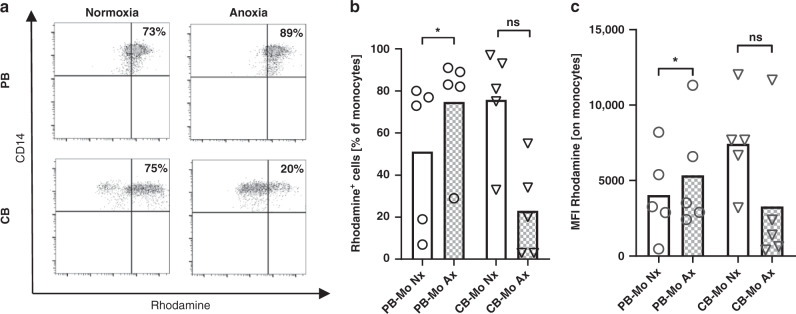
ROS production of cord blood and adult monocytes under anoxia. Peripheral blood mononuclear cells (PBMC) and cord blood mononuclear cells (CBMC) were isolated and cultured under normoxia or under anoxia for four hours. Afterwards, ROS production was detected by Dihydrorhodamine (DHR). **a** Representative density plots show the expression of Rhodamine in CD14^+^ adult monocytes (upper plots) and cord blood monocytes (lower plots) in the upper right quadrant after culture in normoxia (left plots) or anoxia (right plots). **b**, **c** Dot plots with bars show the percentages of Rhodamine^+^ monocytes (**b**) and the mean fluorescent intensity (MFI) for Rhodamine in monocytes (**c**) after culture in normoxia (blank bars) or in anoxia (checked bars). Bars represent pooled data from 5 independent experiments and mean is depicted. *p < 0.05, ns not significant; Wilcoxon matched-pairs signed rank test.

Another important effector function of monocytes is the production of ROS. Thus, we analyzed ROS production of PB and CB monocytes under normoxic and anoxic conditions. As we had observed for phagocytosis, PB monocytes exhibited increased ROS production under anoxia in comparison to normoxia both in percentages of ROS producing monocytes (74.8 ± 25.9% versus 51.2 ± 35.2%, *n* = 5, *p* < 0.05) and in the amount of ROS per cell (MFI 5351 ± 3712 versus 4045 ± 2908, *n* = 5, *p* < 0.05). In CB monocytes ROS production even tended to decrease under anoxia (percentages of ROS producing monocytes 23.0 ± 22.1% versus 75.8 ± 25.5% and MFI 3293 ± 4737 versus 7446 ± 3147, *n* = 5, n.s.) (Fig. 3).

### Adult but not neonatal monocytes show increased phagocytosis capacity upon stimulation with deferoxamine

**Fig. 4:**
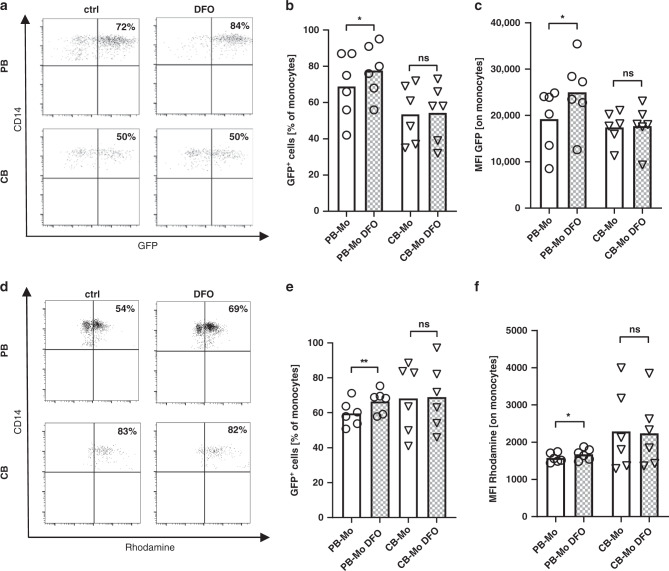
Phagocytosis and ROS production of cord blood and adult monocytes after stimulation with deferoxamine. Peripheral blood mononuclear cells (PBMC) and cord blood mononuclear cells (CBMC) were isolated and cultured under normoxia or under anoxia for four hours. **a**–**c** Afterwards, GFP-expressing *E. coli* (*E. coli*-GFP) were added to the culture for 1 h. **a** Representative density plots show the expression of GFP in CD14^+^ adult monocytes (upper plots) and cord blood monocytes (lower plots) in the upper right quadrant (left plots) without stimulation or after stimulation with deferoxamine (DFO) (right plots). **b**, **c** Dot plots with bars show the percentages of GFP expressing monocytes (**b**) and the mean fluorescent intensity (MFI) for GFP-expression in monocytes (**c**) without stimulation (blank bars) or after stimulation with DFO (checked bars). **d**–**f** ROS production was detected by Dihydrorhodamine (DHR). **d** Representative density plots show the expression of Rhodamine in CD14^+^ adult monocytes (upper plots) and cord blood monocytes (lower plots) in the upper right quadrant without stimulation (left plots) or after stimulation with DFO (right plots). **e**, **f** Dot plots with bars show the percentages of Rhodamine^+^ monocytes (**b**) and the mean fluorescent intensity (MFI) for Rhodamine in monocytes (**c**) without stimulation (blank bars) or after stimulation with DFO (checked bars). Bars represent pooled data from 6 independent experiments and mean is depicted. *p < 0.05, ns not significant; Wilcoxon matched-pairs signed rank test.

To attribute the observed functional differences between PB and CB monocytes under anoxia to altered accumulation of HIF-1α, we next stimulated cells with the HIF-1α stabilizer DFO and analyzed phagocytosis and ROS production. Corresponding to our results under anoxia, we found an increased phagocytic capacity (77.7 ± 14.5% vs. 68.8 ± 17.8% and MFI 24999 ± 7575 vs. 19197 ± 6721, *n* = 6, *p* < 0.05) and an increased ROS production (66.5 ± 6.7% vs. 59.6 ± 7.3% and MFI 1677 ± 149 vs. 1576 ± 123, *n* = 6, *p* < 0.05) in PB monocytes after stimulation with DFO in comparison to unstimulated cells, while phagocytic activity (54.3 ± 15.8% vs. 53.5 ± 16.1% and MFI 17709 ± 4608 vs. 17407 ± 3507, *n* = 6, *p* < 0.05) and ROS production (59.0 ± 18.8% vs. 68.3 ± 19.8% and MFI 2242 ± 930 vs. 2294 ± 1078, *n* = 6, *p* < 0.05) did not change in CB monocytes (Fig. 4).

### Adult monocytes but not neonatal monocytes show increased VEGF production under anoxia

**Fig. 5:**
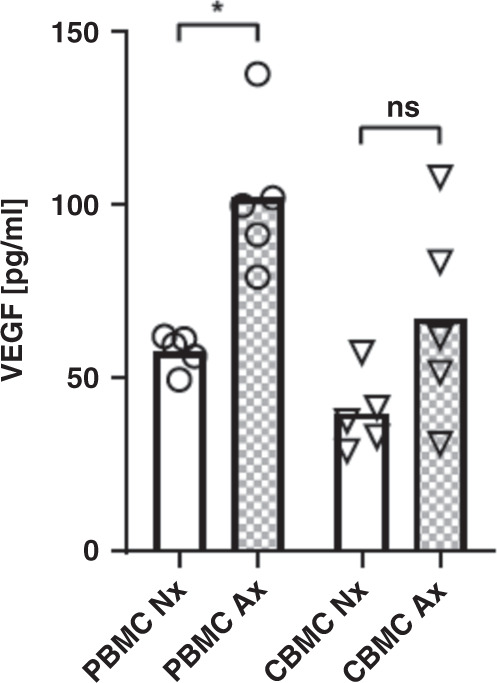
VEGF production of cord blood and adult mononuclear cells under anoxia. Peripheral blood mononuclear cells (PBMC) and cord blood mononuclear cells (CBMC) were isolated and cultured under normoxia or under anoxia for 16–22 h. Expression of VEGF in supernatants was analyzed by ELISA. Dot plot with bars shows VEGF protein in supernatants of adult (white bars) and cord blood (gray bars) mononuclear cells cultured under normoxia (blank bars) or under anoxia (checked bars). Bars represent pooled data from 5 independent experiments and mean is depicted. *p < 0.05; Wilcoxon matched-pairs signed rank test.

Lastly, to analyze a known target effect of HIF-1α expression, we measured expression of VEGF in supernatants of normoxic and anoxic cultured PBMC and CBMC by ELISA. Here, we found increased levels of VEGF in supernatants of PBMC after culture under anoxic in comparison to normoxic conditions (102.0 ± 21.9 pg/ml versus 57.8 ± 5.1 pg/ml, *n* = 5, *p* < 0.05), while VEGF expression in supernatants of CBMC did not increase significantly under culture in anoxia (67.2 ± 29.6 pg/ml versus 39.7 ± 11.0 pg/ml, *n* = 5, n.s.) (Fig. 5). Interestingly, stimulation with *E.coli* did not stimulate but even decreased VEGF production in both PBMC and CBMC (Supplementary Fig. 3).

## Discussion

One of the environmental factors that differs most between fetuses and neonates is their pO_2,_ which is low in the fetus and increases immediately after birth. While the impact of low oxygen pressure on adult immune cells has been well studied, only little is known about how hypoxia/anoxia influence neonatal immune cells. We here investigated the impact of anoxia on immune cells isolated from cord blood in comparison to cells from adult peripheral blood and found that (1) anoxia led to a strong accumulation of protein expression of the transcription factor HIF-1α in adult but not in cord blood immune cells, (2) anoxia induced phagocytosis rates and (3) production of ROS in adult but not in cord blood monocytes, (4) this induction of phagocytosis and ROS production could also be caused by DFO, an inductor of HIF-1α and (4) anoxia led to an increased production of the HIF-1α regulated transcription factor VEGF by adult but not by cord blood immune cells.

Our finding of a lack of upregulation of HIF-1α in neonatal immune cells under anoxia suggests that due to the chronic hypoxia from which the fetus emerges, adaptation processes have taken place that prevent HIF-1α accumulation. Transcription of HIF-1α was affected by anoxia neither in adult nor in cord blood immune cells, pointing towards a post-transcriptional regulation. Ginouvès et al. showed that chronic hypoxia led to an accumulation and overactivation of PHDs and thereby to an induction of HIF-1α protein degradation.^20^ We observed no changes in protein expression of PHD2 between PBMC and CBMC or between normoxic and anoxic culture. However, we did not analyze PHD activity in our samples. Thus, it would be possible that an overactivation of PHDs leads to the reduced actability of HIF-1α in immune cells of the newborn. Furthermore, it has been shown that the mammalian target of rapamycin complex 1 (mTORC1) drives HIF-1α protein accumulation^21^ and that mTOR pathway activation is reduced in neonatal monocytes,^22^ which could be another reason for the lack of HIF-1α expression in these cells.

Interestingly, Ginouvès et al. observed a renewed HIF-1α activation when the extend of hypoxia increased.^20^ This contradicts the results of our study, as we exposed immune cells that came from moderately hypoxic to almost anoxic (<0.1%) conditions. However, Ginouvès’ experiments were done in vitro with periods of chronic hypoxia lasting 3–7 days, so it is perceivable that further adaptation processes take place after very long hypoxia (9 months of a pregnancy in vivo). Furthermore, the experiments of Ginouvès et al. were done with epithelial, fibroblast and cancer cell lines while we examined primary immune cells. Thus, responses to chronic hypoxia may differ between cell types.

It has previously been shown that activation of NF-κB can stimulates transcription of HIF-1α via an NF-κB binding site in the HIF-1α promoter.^11^ Interestingly, in our study stimulation with *E. coli* led to an increase in HIF-1α protein expression as well as HIF-1α mRNA in adult immune cells. In cord blood immune cells, we observed an increase in HIF-1α mRNA, while protein expression could still not be detected. This supports the assumption that post-transcriptional regulation mechanisms, most probably via PHDs, are responsible for the missing HIF-1α protein accumulation in neonatal immune cells.

Next, we found that, consistent with the up-regulation of HIF-1α in adult immune cells, phagocytosis and ROS production were increased under anoxia – an effect that could not be observed in neonatal immune cells. Increased phagocytosis and bacterial killing under hypoxia in a HIF-1α dependent manner have already been described in macrophages from adult mice, macrophage cell lines and human neutrophils.^19,23,24^ Our results of increased phagocytosis and increased ROS production by freshly isolated adult monocytes confirm these results. Several studies have demonstrated that under normal culture conditions phagocytosis of bacteria does not differ between adult and cord blood monocytes.^25,26^ However, phagocytosis of apoptotic cells seems to be diminished in cord blood compared to adult monocytes.^27^ To our knowledge, this is the first study investigating phagocytosis of neonatal monocytes under hypoxic/anoxic conditions. In terms of clinical significance, it could be hypothesized that a lack of ability of neonatal monocytes to increase phagocytosis in the context of hypoxia as occurring in inflamed tissues may have a dual effect. First, it may hint towards a hampered clearance of bacteria during infection, resulting in dissemination of disease. Second, it is possible, that clearance of apoptotic cells, generated during tissue inflammation, may not occur quickly enough, leading to increased secondary necrosis and prolonged inflammation as seen during periventricular leukomalacia (PVL) and bronchopulmonary dysplasia (BPD). Not only during inflammation, but also in the context of systemic hypoxia as seen in asphyxiated newborns, the reduced upregulation of phagocytosis activity in monocytes might be relevant for tissue destruction, as seen during hypoxic–ischemic encephalopathy (HIE).^28,29^

Regarding ROS, it could be shown that ROS increase HIF stability.^30^ Thus, increased ROS production by adult monocytes under hypoxia/anoxia seems to be a positive feedback loop to increase HIF activation. Conversely, the lack of ROS induction in neonatal monocytes probably contributes to the lack of HIF-1α stabilization under anoxia.

We found no upregulation of expression of phagocytosis receptors CD11b and CD18 in adult monocytes upon anoxia but a decrease in both receptors in cord blood monocytes. This does not explain the observed differences in phagocytosis rates in adult and cord blood monocytes under anoxia and is in contrast to studies showing that hypoxia induced expression of CD11b, CD18 and FcγR.^31–33^ Different experimental conditions could underlie these differences. Further studies are needed to elucidate the exact mechanism of the HIF-1-mediated increase in phagocytosis rate seen in adult but not in neonatal monocytes.

In our experiments, stimulation with DFO had the same effect on phagocytosis and ROS production by adult and neonatal monocytes as anoxia had. DFO is an iron chelator that inhibits PHDs and thereby degrades HIF-1α through depleting Fe^2+ ^^34^. This supports the assumption that the observed functional differences between neonatal and adult monocytes are due to reduced HIF-1α activity in neonatal monocytes; but also contradicts the assumption that post-transcriptional regulation of HIF-1α via PHDs is primarily responsible for this effect.

Finally, we showed that anoxia increases the production of VEGF by adult immune cells to a significantly higher extend than seen with neonatal immune cells. As one of the most important known regulators for VEGF expression is HIF-1α,^35^ this supports our hypothesis that HIF-1α cannot be adequately be activated in neonatal immune cells. Interestingly VEGF-expression was even reduced in peripheral blood and cord blood monocytes following stimulation with *E. coli*. The two different HIF-activators tested here, i.e. anoxia and *E.coli*, had different effects. While anoxia did not change HIF-1α mRNA expression, *E.coli* led to an upregulation in both, neonatal and adult immune cells. This is in line with results from Frede et al. showing that PAMPs such as LPS may mediated HIF-1α mRNA induction via NFκ-B activation.^36^

In summary, we could show that there is no activation of the transcription factor HIF-1α under hypoxia in neonatal immune cells, unlike adult immune cells, leading to altered functional properties of particularly of neonatal compared to adult monocytes. Modulation of the reaction of neonatal immune cells to hypoxia (as occurring in the context of inflammation) could be an approach to improve the immune response of the newborn and thereby counteract its increased susceptibility to infection.

## Supplementary information

HIF-WB original

Supplementary Figures

## Data Availability

The datasets generated during and/or analyzed during the current study are available from the corresponding author on reasonable requests.
