# Supplementary figures and images for: Decreased expression of hypoxia-inducible factor 1α (HIF-1α) in cord blood monocytes under anoxia
Source: Pediatr Res. 2022 Jul 29;93(4):870–7. doi: 10.1038/s41390-022-02193-7 (PMC10033401; doi:10.1038/s41390-022-02193-7)

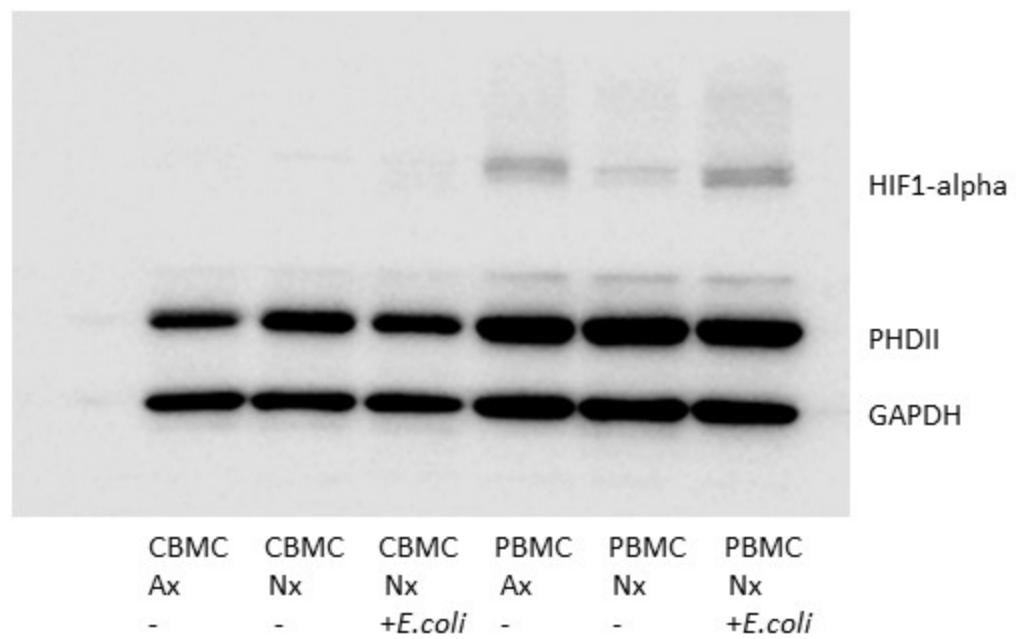

Supplement: Supplementary file 1 — HIF-WB original [file 41390_2022_2193_MOESM1_ESM.pdf]

Supplementary Figure 1

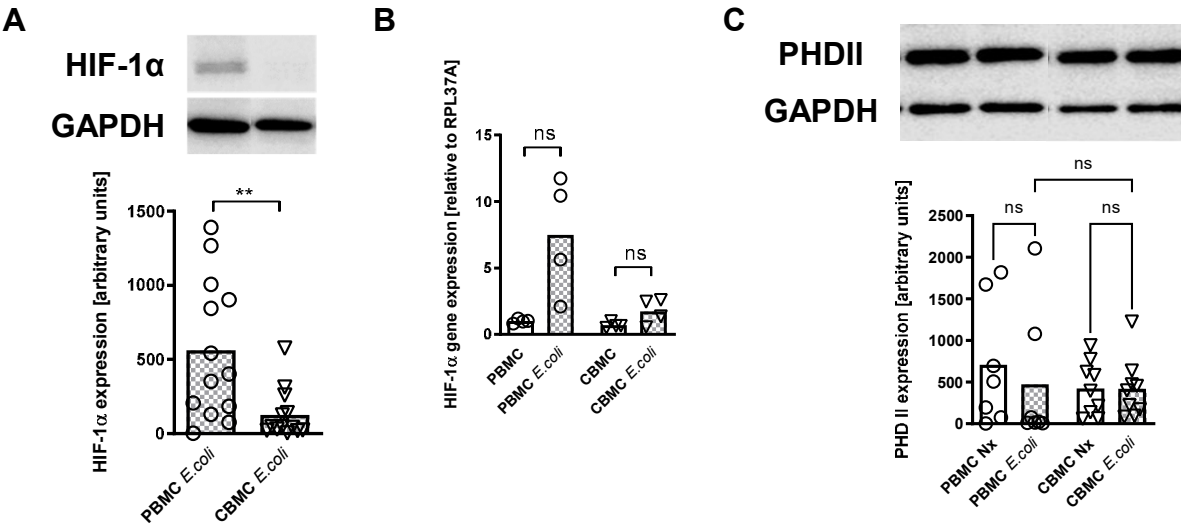

Supplementary Figure 2

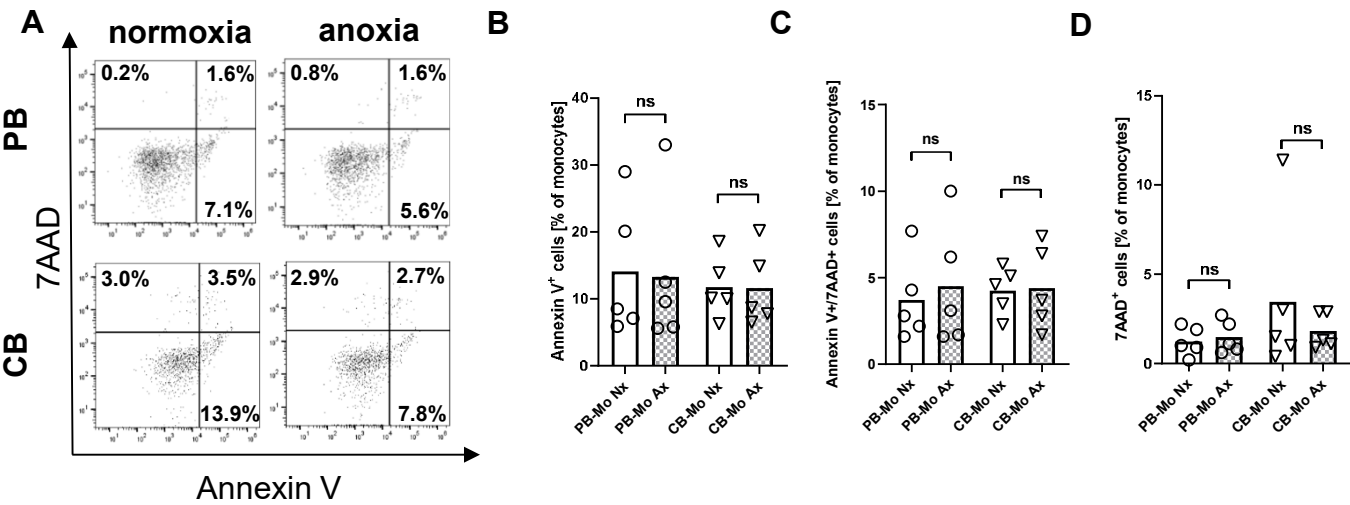

Supplementary Figure 3

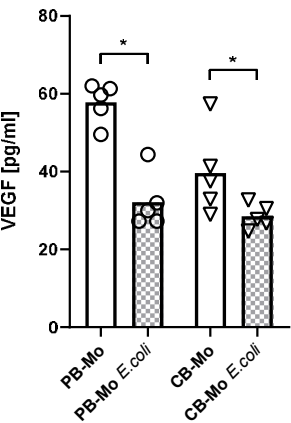

Supplement: Supplementary file 2 — Supplementary Figures [file 41390_2022_2193_MOESM2_ESM.pdf]
